# Cholesterol Efflux Decreases TLR4-Target Gene Expression in Cultured Macrophages Exposed to *T. brucei* Ghosts

**DOI:** 10.3390/microorganisms12081730

**Published:** 2024-08-22

**Authors:** Lawrence Fernando, Jing Echesabal-Chen, Murphy Miller, Rhonda Reigers Powell, Terri Bruce, Apurba Paul, Nava Poudyal, Joshua Saliutama, Kristina Parman, Kimberly S. Paul, Alexis Stamatikos

**Affiliations:** 1 Department of Food, Nutrition, and Packaging Sciences, Clemson University, Clemson, SC 29634, USA; lfernan@g.clemson.edu (L.F.); jchen11@clemson.edu (J.E.-C.); 2 School of Medicine Greenville, University of South Carolina, Greenville, SC 29605, USA; msm8@email.sc.edu; 3 Clemson Light Imaging Facility, Clemson University, Clemson, SC 29634, USA; rhondar@clemson.edu (R.R.P.); terri@clemson.edu (T.B.); 4 Department of Physics and Astronomy, Clemson University, Clemson, SC 29634, USA; 5 Department of Genetics and Biochemistry, Clemson University, Clemson, SC 29634, USA; npoudya@g.clemson.edu (N.P.); kpaul@clemson.edu (K.S.P.)

**Keywords:** apoAI, HDL, kinetoplastid, lipophosphoglycan, LPS, NF-κB, reverse cholesterol transport, variant surface glycoprotein

## Abstract

*Trypanosoma brucei* causes African trypanosomiasis in humans. Infection with *T. brucei* elicits a potent pro-inflammatory immune response within infected human hosts, and this response is thought to at least be partially due to Toll-like receptor (TLR) activation. In response to stimulation by lipopolysaccharide and other pathogen antigens, TLR4 translocates to lipid rafts, which induces the expression of pro-inflammatory genes. However, cholesterol efflux is acknowledged as anti-inflammatory due to promoting lipid raft disruption. In this study, we wanted to assess the impact of *T. brucei* “ghosts”, which are non-viable *T. brucei* essentially devoid of intracellular contents, in stimulating macrophage TLR4 translocation to lipid rafts, and whether promoting cholesterol efflux in macrophages incubated with *T. brucei* ghosts attenuates TLR4-target gene expression. When cultured macrophages were exposed to *T. brucei* ghosts, we observed an increase in lipid raft TLR4 protein content, which suggests certain surface molecules of *T. brucei* serve as ligands for TLR4. However, pretreating macrophages with cholesterol acceptors before *T. brucei* ghost exposure decreased lipid raft TLR4 protein content and the expression of pro-inflammatory TLR4-target genes. Taken together, these results imply that macrophage cholesterol efflux weakens pro-inflammatory responses which occur from *T. brucei* infection via increasing macrophage lipid raft disruption.

## 1. Introduction

*Trypanosoma brucei* is a eukaryotic pathogen that causes African sleeping sickness in humans and nagana in livestock [1]. African sleeping sickness and nagana are vector-borne diseases because transmission occurs when an infected tsetse fly bites a mammalian host [2]. African trypanosomiasis is considered a neglected tropical disease, as tsetse flies are only thought to inhabit sub-Saharan Africa [3]. If left untreated, African trypanosomiasis is generally fatal [4]. However, conventional treatments for African trypanosomiasis are prone to side effects [5]. Indeed, complications from traditional types of therapeutic interventions for African trypanosomiasis have been reported to be severe enough to becoming life-threatening, and in some cases, even fatal [6,7].

African trypanosomiasis is well-recognized to be endemic to sub-Saharan Africa [8]. While improved screening and increased treatment rates have reduced the number of reported cases for human African trypanosomiasis, this disease is still considered a serious concern in rural sub-Saharan African areas stricken by poverty, since African sleeping sickness often goes under-reported within these regions [3]. African trypanosomiasis is also a significant burden for the veterinary and agricultural industry. It is estimated that between 50 and 55 million cattle are at risk for developing nagana and approximately 3 million cattle die yearly from this disease [9,10]. Animal African trypanosomiasis also drastically afflicts sheep and goats, as approximately 30 million sheep and an estimated 40 million goats are at risk of being infected annually [9,10]. Furthermore, without prompt and effective treatments, African trypanosomiasis in livestock will likely lead to mortality [11,12].

African trypanosomiasis leads to a strong host pro-inflammatory immune response that attempts to clear *T. brucei* from the body [13]. However, *T. brucei* is able to escape host immunity by undergoing antigenic variation via variant surface glycoprotein (VSG) switching, allowing *T. brucei* to persist within infected mammalian hosts [14,15,16]. Therefore, it is not entirely clear what benefit pro-inflammatory immune responses play during *T. brucei* infection, and some evidence has suggested that this type of host response may be deleterious to infected hosts [17,18]. It is also poorly understood what mechanisms and processes activate the pro-inflammatory immune response from *T. brucei* infection [19,20]. *T. brucei* is associated with certain surface molecules [21] that appear capable of acting as ligands for different Toll-like receptors (TLRs). For instance, data suggest that VSG, which is the most abundant surface molecule of bloodstream form *T. brucei*, may demonstrate the capacity to induce various TLRs [21,22,23,24]. Data have also shown that lipophosphoglycan, which is a minor surface component of *T. brucei*, serves as a ligand for both TLR2 and TLR4 [25,26]. Thus, TLR activation mediated by *T. brucei* surface molecules may at least partially explain how *T. brucei* parasites stimulate inflammation within infected hosts.

Early in *T. brucei* infection, macrophages in the liver and spleen mediate the humoral clearance of parasites and influence the developing immune response [27,28]. Macrophages contain a high lipid raft content and also exhibit potent pro-inflammatory effects from lipopolysaccharide (LPS) exposure [29]. Moreover, macrophages are known to efficiently efflux cholesterol to apoAI and HDL via the cholesterol efflux transporters ABCA1 and ABCG1, respectively [30]. We and others have shown that ABCA1/apoAI- and ABCG1/HDL-mediated cholesterol efflux decreases pro-inflammatory gene expression in macrophages and other cells that are challenged with the canonical TLR4 ligand LPS or other TLR4 ligands, and these effects are from cholesterol-efflux-reducing lipid raft TLR4 protein content [31,32,33,34,35,36,37]. Regarding TLRs, the TLR which is of central importance to innate immunity is TLR4 [38,39]. Interestingly, for TLR4 to induce pro-inflammatory gene expression, TLR4 first needs to translocate to lipid rafts after being activated by ligands on the cell surface [40]. Furthermore, the removal of cholesterol from cells promotes lipid raft disruption, and so cellular cholesterol efflux has been acknowledged as being anti-inflammatory due to stimulating lipid raft disruption [41]. Macrophages are also well-established to play a critical role in the pro-inflammatory immune response that occurs during *T. brucei* infection [42,43].

Hence, the purpose of our work was to assess whether apoAI/HDL-mediated cholesterol efflux in macrophages is capable of attenuating the pro-inflammatory response that occurs when these cells are exposed to *T. brucei*. In this work, we aimed to analyze the capacity of the entire cell surface of *T. brucei* to act as a ligand for TLR4 within cultured macrophages. In this work, when we utilized non-viable *T. brucei* “ghosts” and incubated macrophages with these parasite ghosts (PGs), this resulted in increased TLR4 translocation to lipid rafts within the cultured macrophages. However, pretreating cultured macrophages with the cholesterol acceptors apoAI or HDL before challenging these cells with PG decreased TLR4 translocation to lipid rafts. Furthermore, macrophage apoAI/HDL pretreatment also resulted in reducing the pro-inflammatory expression of TLR4-target genes in macrophages incubated with PG. Based on our results, we conclude that apoAI- and HDL-mediated cholesterol efflux is capable of attenuating TLR4-target gene expression in macrophages exposed to *T. brucei* and that this effect is likely due to promoting macrophage lipid raft disruption which decreases TLR4 translocation to lipid rafts.

## 2. Materials and Methods

### 2.1. Cell Culture and Maintenance of Cells

We purchased RAW 264.7 macrophage cells [44] from ATCC (Manassas, VA, USA) and maintained this cell line in tissue culture plates and standard growth medium that contained high-glucose Dulbecco’s modified Eagle’s medium (DMEM; Corning, New York, NY, USA), fetal bovine serum (FBS) (10%; VWR Life Science Seradigm, Radnor, PA, USA), and penicillin-streptomycin (1%; Corning). For this cell line, we replenished the medium approximately biweekly; once cells reached 70–90% confluency, they were either passaged into maintenance tissue culture plates or plated to treatment dishes. Bloodstream form *T. brucei brucei* Lister 427 strain (BF-427) were cultured within flasks using HMI 9 medium [45] supplemented with 10% heat-inactivated FBS and 1% pencillin-streptomycin. We sub-cultured BF-427 every 2–3 days until cells were ready for their respective treatments. Both types of cells were grown and maintained in an incubator set at 37 °C and 5% CO_2_.

### 2.2. Preparation and Characterization of BF-427 Parasite Ghosts (PGs)

We prepared BF-427 PG by adapting methods as previously described [25]. We exposed cultured BF-427 to a working concentration of 1% saponin (Sigma-Aldrich, St. Louis, MO, USA) for 15 min at 4 °C. After saponin treatments, we then either assessed PG via fluorescent microscopy, or collected PG via centrifugation, washed the pelleted PG with phosphate-buffered saline (PBS), repeated centrifugation, and then removed PBS and resuspended PG to utilize for downstream experiments.

We utilized a rat anti-“Tryps” primary antibody to aid in visualizing the PG. To create the anti-Tryps primary antibody, male retired breeder CD1 rats (Charles River, Wilmington, MA, USA) were infected intra-peritoneally (i.p.) with 5 × 10^5^ *T. brucei* in sterile phosphate-buffered saline (PBS). When parasitemia reached 0.5 × 10^8^, rats were cured with two doses of diminazene [46] (34.1 mg/kg i.p.; Cayman Chemical, Ann Arbor, MI, USA) administered 24 h apart. After 20 days to allow for the development of a mature antibody response, blood from cured rats was collected by cardiac puncture and allowed to clot, and the serum supernatant was stored at −80 °C. Serum proteins were concentrated by ammonium sulfate precipitation, and serum IgMs were affinity-enriched with an IgM Purification Kit (Pierce Biotechnology/Thermo Scientific, Rockford, IL, USA) using the manufacturer’s instructions, except ammonium sulfate precipitate was dialyzed into 20 mM Tris-Cl (pH 7.4), 1.25 M NaCl. The serum IgGs were then enriched by passing the IgM column flow-through (which contained the IgGs and other serum proteins) over a Pierce Melon Gel IgG purification kit (Pierce Biotechnology/Thermo Scientific) using the manufacturer’s instructions to remove serum albumin and other contaminating serum proteins. The resulting affinity-enriched IgG antibody was used then used to visualize the PG.

To stain the PG surface for imaging, we plated prepared PG on poly-L-lysine coated slides and allowed PG to settle for 20 min at room temperature. Residual liquid was removed and then PG were fixed for 1 h in 2% paraformaldehyde at room temperature in a humid chamber. PG were washed 4X in PBS, permeabilized with 0.1% triton-X-100/PBS for 20 min, and then washed again with PBS. We incubated PG in block solution (10% goat serum/0.1% triton-X-100/PBS) for 1 h at room temperature, followed by an overnight, 4 °C incubation with the rat anti-”Tryps” primary antibody (diluted 1:10). Following incubation with this primary antibody, PG were washed 3X in wash solution, incubated in Alexa Fluor 488 goat anti-rat IgG secondary antibody (Invitrogen, Carlsbad, CA, USA), which was diluted 1:200, washed again 3X using wash solution, and then incubated with DAPI (Invitrogen) [47]. Following incubation with DAPI counterstain, PG were washed twice with wash solution, and then mounted in ProLong Gold. We imaged PG with a Leica SP8X confocal microscope (Leica Microsystems, Buffalo Grove, IL, USA) equipped with a 405 nm laser, a tunable white light laser (WLL), HyD detectors, and time gating. To detect DAPI, PG were excited using the 405 nm laser, and emission wavelengths of 410–460 nm were collected by using a HyD detector. For antibody detection, PG were excited using the WLL tuned to 499 nm, and emission wavelengths of 510–550 nm were collected by using the HyD detector with a 0.5 ns time gate. The PG were imaged using a 100X/1.40 N.A. oil immersion lens with 1.5X zoom, and images were collected with using Leica LAS X software (Version 3.5.7.23225, Leica Microsystems, Buffalo Grove, IL, USA) and exported as .TIF image files.

### 2.3. Macrophage Exposure to PG

We pre-incubated cultured macrophages in serum-free DMEM containing 1% pencillin-streptomycin and 2 mg/mL of fatty acid-free bovine serum albumin (Sigma-Aldrich) [48]. To intially assess TLR4 translocation to lipid rafts in cultured macrophages exposed to PG, we treated cells using these cultured conditions and incubated macrophages with either vehicle only or PG (1:1 PG to macrophage ratio) for 24 h, washed cells with PBS, and then collected the non-raft and lipid raft fractions from macrophages. To promote lipid raft disruption, we first exposed macrophages to either 50 μg/mL apoAI (Academy Bio-Medical Company, Houston, TX, USA), 50 μg/mL HDL (Academy Bio-Medical Company), or vehicle only during serum-free DMEM culturing conditions [49,50]. After 24 h of cholesterol acceptor and vehicle treatments, we removed the medium and washed macrophages with PBS, then challenged cells with PG in serum-free DMEM. After 24 h of PG treatment, we washed cells with PBS and either isolated the non-raft and lipid raft fractions from macrophages or harvested total RNA from the treated cells.

### 2.4. Isolation of Macrophage Lipid Rafts and Immunoblotting

To isolate non-raft and lipid raft fractions from macrophages, we utilized a detergent-free, gradient ultracentrifugation method [36] successfully used by us previously [33]. Briefly, we spun macrophage lysates at 50,000 rpm maximum speed for 24 h at 4 °C by using an Optima MAX-XP ultracentrifuge (Beckman Coulter, Brea, CA, USA) and an MLS-50 swinging bucket rotor (Beckman Coulter). After ultracentrifugation, we collected 9 equal volume fractions, discarded the fifth fraction, then pooled equal volumes from the 4 remaining lipid-raft fractions and 4 remaining non-raft fractions. We separated proteins from the pooled fractions using SDS-PAGE and then transferred the separated proteins onto PVDF membranes [51]. After incubating PVDF membranes in the blocking buffer, we incubated the membranes overnight with either mouse anti-TLR4 primary antibody (1:1000 dilution, sc-293072; Santa Cruz Biotechnology, Dallas, TX, USA) or rabbit anti-caveolin-1 primary antibody (1:750 dilution; 3267, Cell Signaling Technology, Danvers, MA, USA). After primary antibody incubation and washing steps, we incubated the PVDF membranes with either HRP-conjugated goat anti-mouse IgG secondary antibody (1:10,000 dilution, AP181P; Sigma-Aldrich) or HRP-conjugated goat anti-rabbit IgG secondary antibody (1:10,000 dilution, HAF008; Novus Biologicals, Littleton, CO, USA). To visualize proteins, we utilized an ECL-based detection and imaging ChemiDoc instrument (Analytik Jena US, Upland, CA, USA) [52].

### 2.5. Macrophage Total RNA Extraction and RT-qPCR

We isolated total RNA from PG-challenged macrophages as previously described [53]. Briefly, we initially lysed macrophages with TRIzol and then extracted total RNA from lysed macrophages by using a Direct-Zol RNA purification kit (Zymo Research, Irvine, CA, USA). We then quantified the purified total RNA using a SpectraMax^®^ QuickDrop™ Micro-Volume Spectrophotometer (Molecular Devices, LLC., San Jose, CA, USA). Using equal mass of RNA per RNA sample, we converted the total RNA into cDNA with Quantabio qScript Ultra SuperMix reagent (Beverly, MA, USA). We used the newly synthesized cDNA for qPCR reactions by utilizing a Quantabio PerfeCTa SYBR Green Fastmix kit along with the following primer pairs to amplify genes of interest and GAPDH housekeeping gene: IL-1β (forward primer, 5′-GAAATGCCACCTTTTGACAGTG-3′; reverse primer, 5′-TGGATGCTCTCATCAGGACAG-3′); IL-6 (forward primer: 5′-TCTATACCACTTCACAAGTCGGA-3′; reverse primer: 5′-GAATTGCCATTGCACAACTCTTT-3′); TNF-α (forward primer: 5′-CAGGCGGTGCCTATGTCTC-3′; reverse primer: 5′-CGATCACCCCGAAGTTCAGTAG-3′); GAPDH internal reference for normalization (forward primer: 5′-CGTGCCGCCTGGAGAAAC-3′; reverse primer: 5′-TGGGAGTTGCTGTTGAAGTCG-3′) [33,54]. The amplified qPCR reactions were quantified with a qTOWER^3^ G touch qPCR instrument (Analytik Jena US) [55], and gene expression was analyzed by following the delta-delta CT (ΔΔ^CT^) method [56].

### 2.6. Statistical Analysis

To perform statistical analyses, we utilized SigmaPlot (v14.0) software (Systat Software Inc., San Jose, CA, USA). To determine the appropriateness of conducting Student’s *t*-test, we initially performed Brown–Forsythe and Shapiro–Wilk tests to assess equal variance and normality, respectively. When the Brown–Forsythe test failed, we performed Welch’s *t*-test. When the Shapiro–Wilk test failed, we conducted a Mann–Whitney rank-sum test. We set the level of significance for experiments requiring statistical analysis at *p* < 0.05.

## 3. Results

### 3.1. Exposing Cultured Macrophages to T. brucei “Ghosts” Triggers TLR4 Translocation to Lipid Rafts

**Figure 1:**
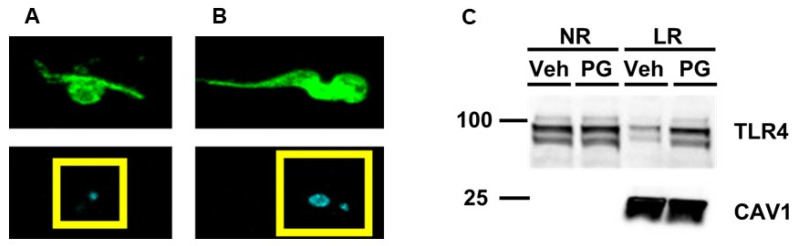
Enrichment of TLR4 within the lipid raft fractions of cultured macrophages challenged with PG. Immunofluorescence staining of a representative BF-247 PG (**A**) and viable BF-427 parasite (**B**) for detecting the cell surface of BF-427 (green in top two panels) and nuclei (blue/cyan in bottom two panels). Yellow boxes enclose nuclear region showing only mitochondrial kinetoplast DNA (kDNA) present in the BF-427 PG (**A**), while both the nucleus and kDNA were detected in viable BF-427 based on DAPI counterstain. (**C**) Cultured macrophages were incubated with either vehicle only (Veh) or PG and pooled non-raft (NR) and lipid raft (LR) fractions were collected from macrophages to probe for TLR4 and the lipid raft marker caveolin-1 (CAV1) using immunoblotting. Size markers are in kDa.

The host pro-inflammatory immune response against *T. brucei* is complex [18,57]. For instance, *T. brucei* is capable of activating TLR9, and data have shown that the intracellular DNA of *T. brucei* activates this TLR [23,58,59]. Moreover, TLR9 is an endosomal TLR, unlike cell surface TLR4 [60,61]. Therefore, to inhibit the intracellular components of *T. brucei* stimulating inflammation within cultured macrophages, we generated non-viable BF-427 PG, which results in the removal of intracellular contents from these parasites while still preserving BF-427 surface molecules [25]. Using immunofluorescent staining, we confirmed we were capable of generating PG (Figure 1A,B). We utilized these PGs to treat cultured macrophages to initially assess whether exposing macrophages to PG stimulates TLR4 translocation to lipid rafts. When we used immunoblotting to assess TLR4 protein levels, we observed an increase in TLR4 protein content within the lipid raft fractions of cultured macrophages challenged with PG when compared to control macrophages treated with vehicle only (Figure 1C). Based on this result, we concluded that the cell surface of *T. brucei* functions as a ligand for TLR4.

### 3.2. Pretreating Cultured Macrophages with Cholesterol Acceptors Reduces PG-Induced TLR4 Translocation to Lipid Rafts

**Figure 2:**
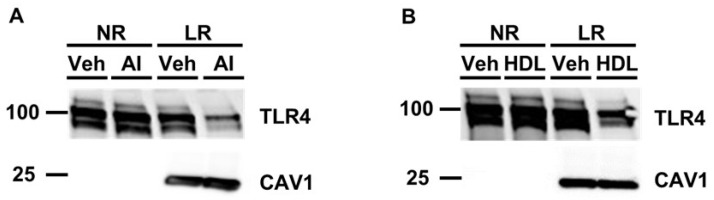
Exposing cultured macrophages to apoAI or HDL before PG stimulation decreases TLR4 translocation to lipid rafts. (**A**,**B**) Cultured macrophages were either pre-incubated with vehicle only (Veh), apoAI (“AI” shown in panel **A**), or HDL (**B**) before being challenged with PG. Pooled non-raft (NR) and lipid raft (LR) fractions were collected from treated macrophages to probe for TLR4 and the lipid raft marker caveolin-1 (CAV1) via immunoblotting. Size markers are in kDa.

Cellular lipid raft disruption occurs when cholesterol is removed from cells, and we and others have shown that apoAI- and HDL-mediated cholesterol efflux is a robust method to promote lipid raft disruption [33,35,36]. Thus, we wanted to analyze whether pretreating cultured macrophages with the cholesterol acceptors apoAI or HDL decreases TLR4 translocation to lipid rafts when macrophages later become exposed to PG. In cultured macrophages that were first incubated with either apoAI (Figure 2A) or HDL (Figure 2B) before being challenged with PG, we observed a reduction in TLR4 translocation to lipid rafts when compared to the respective vehicle control pretreated macrophages later incubated with PG. Since apoAI acts as the exclusive cholesterol acceptor for ABCA1, and ABCG1 can participate in cholesterol efflux via interacting with HDL [30], we concluded from these findings that ABC-transporter-dependent cholesterol efflux is able to decrease TLR4 translocation to lipid rafts in macrophages exposed to PG through stimulating lipid raft disruption.

### 3.3. ApoAI- and HDL-Mediated Cholesterol Efflux Decreases TLR4-Target Gene Expression in Cultured Macrophages Exposed To PG

**Figure 3:**
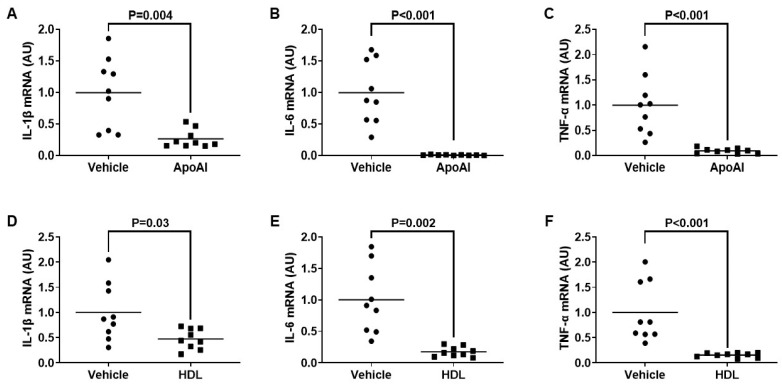
Pretreating cultured macrophages with cholesterol acceptors attenuates TLR4-target gene expression induced by PG exposure. (**A**–**F**) TLR4 target genes IL-1® (**A**,**D**), IL-6 (**B**,**E**), and TNF-〈 (**C**,**F**) were measured in control macrophages pretreated with vehicle only (**A**–**F**), or macrophages pretreated with either apoAI (**A**–**C**) or HDL (**D**–**F**), before all macrophages were incubated with PG. (**A**–**F**) Gene expression measured via qRT-PCR and AU = arbitrary units. Data points are from three independent treatments that include three biological replicates for each respective treatment. Bars are group means.

Our team and other laboratories have shown that if LPS-mediated TLR4 translocation to lipid rafts becomes inhibited, then this attenuates the expression of pro-inflammatory TLR4-target genes [33,34,35]. Hence, in this study, we wanted to assess if macrophage cholesterol efflux may reduce the expression of TLR4-target genes in these cells when they are exposed to PG. Using RT-qPCR, we measured mRNA expression of the well-established TLR4-target genes IL-1β, IL-6, and TNF-α [62] in macrophages challenged with PG and either pretreated with the cholesterol acceptors apoAI and HDL or pretreated with vehicle only. In these experiments, we observed a significant decrease in the expression of all pro-inflammatory genes within the cultured macrophages pretreated with the cholesterol acceptors when compared to the respective vehicle control pretreatment groups (Figure 3). From these results, we conclude that incubating macrophages with apoAI and HDL attenuates the inflammatory response which normally occurs in these cells upon exposure to *T. brucei* and that this effect is likely due to ABCA1/apoAI- and ABCG1/HDL-mediated cholesterol efflux promoting lipid raft disruption which reduces macrophage TLR4 translocation to lipid rafts.

## 4. Discussion

**Figure 4:**
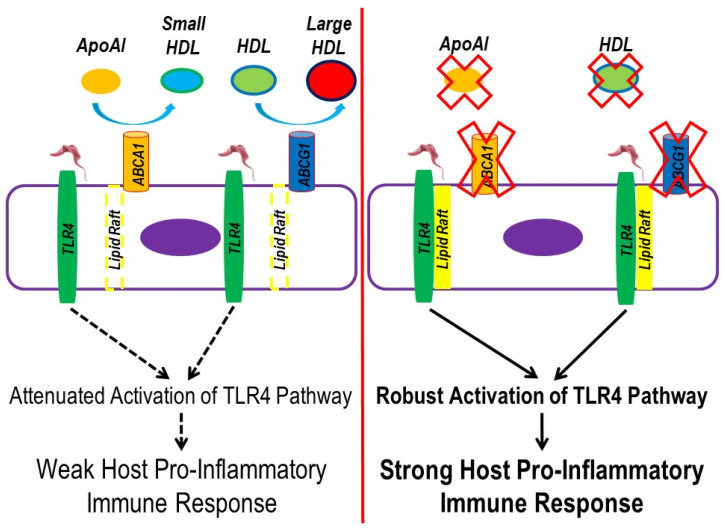
Working model to propose ABC-transporter mediated cholesterol efflux reduces the pro-inflammatory immune response that initially occurs from *T. brucei* infection. (**Left panel**) Functional ABCA1 and ABCG1 efflux cholesterol upon interaction with apoAI and HDL, which promotes lipid raft disruption. During *T. brucei* infection, ligands on the parasite surface bind to macrophage surface TLR4. However, TLR4 translocation to lipid rafts becomes minimized upon enhanced macrophage ABCA1/apoAI- and ABCG1/HDL-mediated cholesterol efflux. The reduced abundance of lipid rafts in macrophages attenuates activation of the TLR4 signaling pathway, weakening the host pro-inflammatory immune response that normally occurs in *T. brucei* infection. (**Right panel**) Impaired ABCA1/apoAI- and ABCG1/HDL-mediated cholesterol efflux in macrophages stabilizes lipid rafts within macrophages, enhancing TLR4 translocation and causing robust activation of the TLR4 signaling pathway in response to *T. brucei* ligands, which results in a strong host pro-inflammatory immune response.

In our study, we wanted to assess a possible pro-inflammatory impact that the cell surface of *T. brucei* may induce in macrophages and if this potential pro-inflammatory response is regulated by TLR4. To capture the entire cell surface landscape of *T. brucei*, we prepared PG from BF-427 so that parasite viability and the intracellular contents of *T. brucei* may be controlled when analyzing inflammation in cultured macrophages. When we exposed cultured macrophages to PG, we observed an increase in TLR4 translocation to lipid rafts within these cells, which strongly suggests certain cell surface components of *T. brucei* serve as a ligand for TLR4. Because TLR4 translocation to lipid rafts is required to robustly trigger a pro-inflammatory immune response [63], we then wanted to directly test whether apoAI- and HDL-mediated cholesterol efflux can confer protection against inflammation within macrophages exposed to PG via reducing TLR4 translocation to lipid rafts, as ABCA1/apoAI- and ABCG1/HDL-mediated cholesterol efflux enhances lipid raft disruption [64]. When macrophages were incubated with either apoAI or HDL before PG exposure, we did observe decreased macrophage TLR4 translocation to lipid rafts, which indicates apoAI- and HDL-mediated cholesterol efflux reduces TLR4 translocation to lipid rafts via promoting lipid raft disruption. Furthermore, when we measured the expression of the TLR4-target genes IL-1β, IL-6, and TNF-α in these same conditions, we detected decreased mRNA expression of all pro-inflammatory TLR4-target genes within the cultured macrophages pretreated with either apoAI or HDL when compared to respective control macrophages pretreated with vehicle only. Taken together, these results suggest ABCA1/ABCG1-dependent cholesterol efflux within macrophages reduces the pro-inflammatory response that occurs when becoming infected with *T. brucei*. If so, then cholesterol efflux could be considered a potential novel host anti-inflammatory process occurring during *T. brucei* infection (Figure 4).

Though it is well-recognized that infection with *T. brucei* induces pro-inflammatory immune responses within infected hosts, the processes that govern these responses in hosts are complicated and not entirely well-defined. For instance, while data have shown that the spleen and other cells of the reticuloendothelial system play a large role in modulating the host pro-inflammatory during *T. brucei* infection [65], emerging evidence has shown that adipocytes exhibit a robust pro-inflammatory immune response in hosts infected with *T. brucei* [66]. This is intriguing, as adipose tissue has traditionally been thought to be a mostly benign tissue with low hormonal activity, even during times of illness and infection [67,68,69,70,71,72,73]. Other data have observed that the brain and central nervous system, lungs, skin, and reproductive system are intricately related to the immune response that mounts in hosts during *T. brucei* infection [74,75,76,77,78,79,80,81,82,83,84,85,86]. With so many cell and tissue types being at least indirectly involved with the pro-inflammatory immune response that occurs from *T. brucei* infection, it is very likely that there are numerous inflammatory and immune factors also present during infection with *T. brucei*. It is already recognized that an innate immune response occurs during *T. brucei* infection and that this effect may be TLR-mediated [23,25,26,87]. In this study, we clearly show that non-viable PG are effective in triggering a pro-inflammatory response within cultured macrophages by acting as a ligand for TLR4, which may partially explain how *T. brucei* activates the innate immune system in infected mammals.

Therapies for human African trypanosomiasis vary based on whether infection has been restricted to the hemolymphatic system, which is known as the first stage of infection, or whether the disease has advanced via parasites crossing the blood–brain barrier to reach the host brain and central nervous system, which is defined as the second meningoencephalitis phase [88]. The second phase is notorious to treat effectively due to certain traditional therapies being extremely toxic to the hosts, sometimes leading to fatality [3,6,88,89]. Although more novel polytherapies have been developed (e.g., nifurtimox-eflornithine combination therapy), the therapeutic regimen of these types of treatments is very complicated, requiring intensive training, labor, and resources for appropriate administration [90,91]. For newer therapies to combat African trypanosomiasis, it is important to note that some treatments may be contraindicated in certain patients, as well as being considered to be cost-prohibitive [88,92,93,94,95,96,97,98,99,100]. Moreover, there are serious potential issues about drug resistance with newer types of treatments for human African trypanosomiasis [95,97,101,102]. Interestingly, the mechanisms of actions for treatments involving African trypanosomiasis are diverse [88], but none appear to directly influence cellular cholesterol efflux and metabolism. While it is not currently known if manipulating cell cholesterol content in hosts infected with *T. brucei* is either beneficial or harmful to combating disease progression, there is evidence that suggests infectivity with the trypanosomatid parasite *Lelishmania donovani* depends on host–cell cholesterol metabolism, though these data are convoluted. Indeed, the pathogenesis of *L. donovani* appears to be enhanced when cells contain high levels of cholesterol, but after disease initiation and especially during the later stages of *L. donovani* infection, virulence seems to become advanced when host cellular cholesterol becomes depleted [103,104,105,106,107,108,109,110,111]. If cellular cholesterol efflux does impact the pro-inflammatory immune response that occurs within infected hosts during *T. brucei* infection via inhibiting TLR4-target gene expression through lipid raft disruption, then a similar quandary may exist for host–cell cholesterol metabolism influencing *T. brucei* pathogenicity. It has been proposed that the initial pro-inflammatory immune response against *T. brucei* is beneficial, but in the more advanced stages of disease, this response is considered to be deleterious [58,85,86,112,113].

There are limitations in our study. One main limitation is using the RAW 264.7 mouse macrophage cell line in our experiments instead of utilizing primary macrophages. While we have experience working with this macrophage cell line, and RAW 264.7 macrophages contain lipid rafts, robustly express ABCA1 and ABCG1, efflux cholesterol to both apoAI and HDL, and demonstrate a strong pro-inflammatory response to TLR4 ligands such as LPS [44,114,115,116,117], we acknowledge that data generated from immortalized cells may not be comparable to primary cells, as immortalized cell lines sometimes lack similar characteristics and properties as primary cells [118]. Therefore, future studies should be conducted to confirm that the cell surface of *T. brucei* does serve as a ligand for TLR4 in primary macrophages. Another major limitation of our study is not directly testing the potential pro-inflammatory effects which occur in cultured macrophages exposed to PG that are not mediated by TLR4. A strong strategy to test for this in the future is to utilize TLR4 knockout macrophages derived from TLR4 knockout mice [119,120]. It is certainly possible that the cell surface of *T. brucei* may also robustly induce the activation of cell surface TLR2 and respective pro-inflammatory genes, which would be a novel finding. In this scenario, future studies should also employ TLR2 knockout macrophages derived from TLR2 knockout mice [119,121] to gauge the pro-inflammatory impact of TLR4 versus TLR2 activation and induction of their respective pro-inflammatory target genes in cultured macrophages exposed to PG. This would enable an assessment of what surface TLR is the main driver in stimulating macrophage inflammation upon exposure to *T. brucei* surface ligands. Lastly, both apoAI and HDL have been proposed to exhibit anti-inflammatory effects that are independent of their contribution to lipid raft disruption via cholesterol efflux. Therefore, future studies should also be conducted by utilizing ABCA1 and ABCG1 knockout macrophages [122] to directly analyze what possible anti-inflammatory effects occur within cultured macrophages exposed to PG that have been pretreated with the cholesterol acceptors apoAI and HDL, as the ablation of ABCA1/ABCG1 would prevent apoAI/HDL-mediated cholesterol efflux.

## 5. Conclusions

Our study shows that incubating macrophages with either apoAI or HDL attenuates pro-inflammatory TLR4-target gene expression that is stimulated from *T. brucei* exposure and that this effect is likely due to ABCA1- and ABCG1-dependent cholesterol efflux. Future studies should focus on which precise surface molecule(s) of *T. brucei* are responsible for TLR4 activation and whether manipulating apoAI/HDL levels or ABC-transporter expression in animal models infected with *T. brucei* impacts pro-inflammatory immune responses within these hosts. If modulating cholesterol efflux does alter both pro-inflammatory immune response and parasite load, then possible treatments for African trypanosomiasis may include therapies that can alter apoAI/HDL plasma levels and/or change cellular ABC-transporter expression [44,53,55,123,124,125,126,127], as efficacy for these types of proposed treatments can easily be analyzed within a pre-clinical setting.

## Data Availability

All data that are represented within this study are contained in the manuscript.
